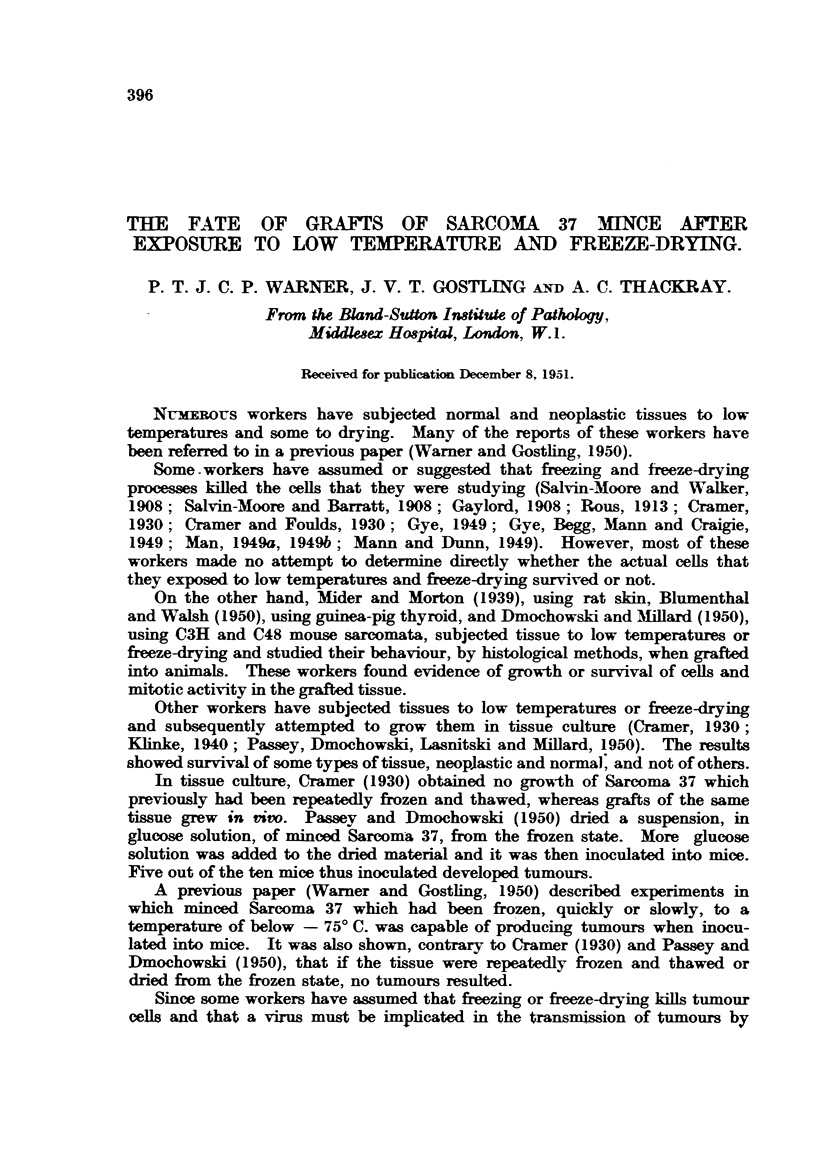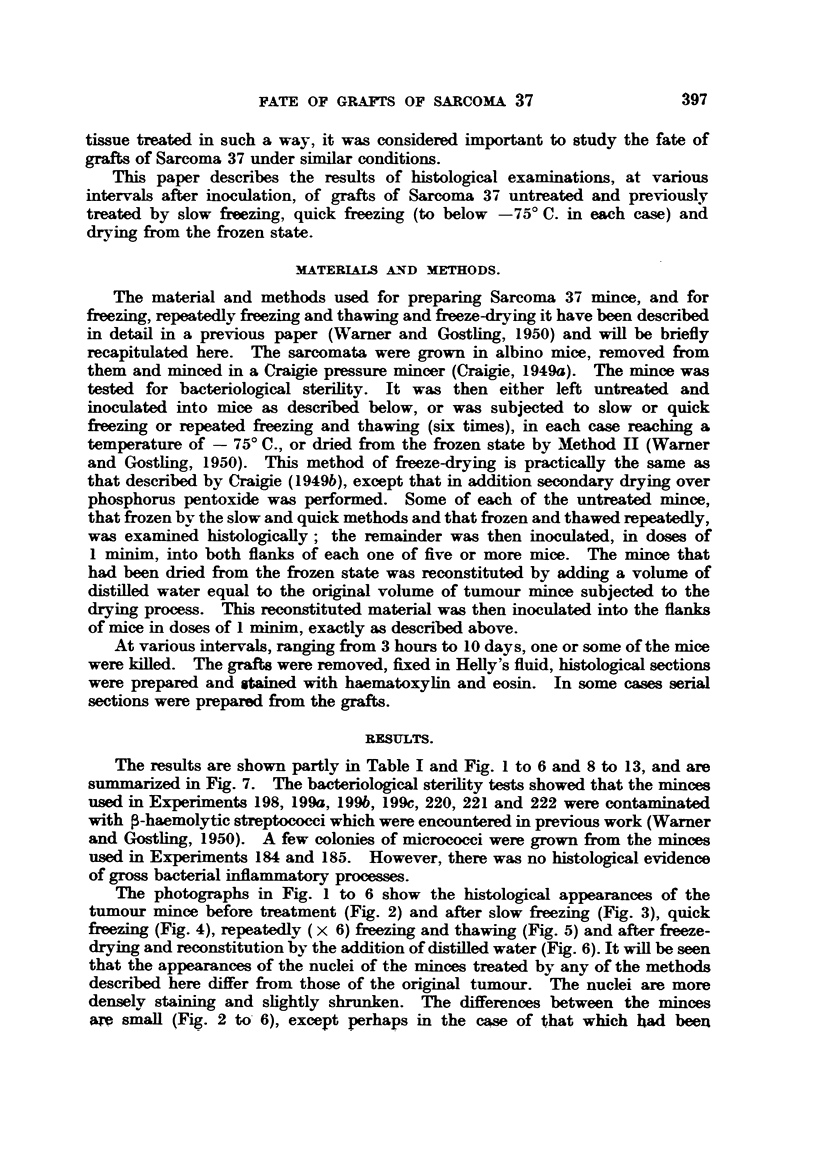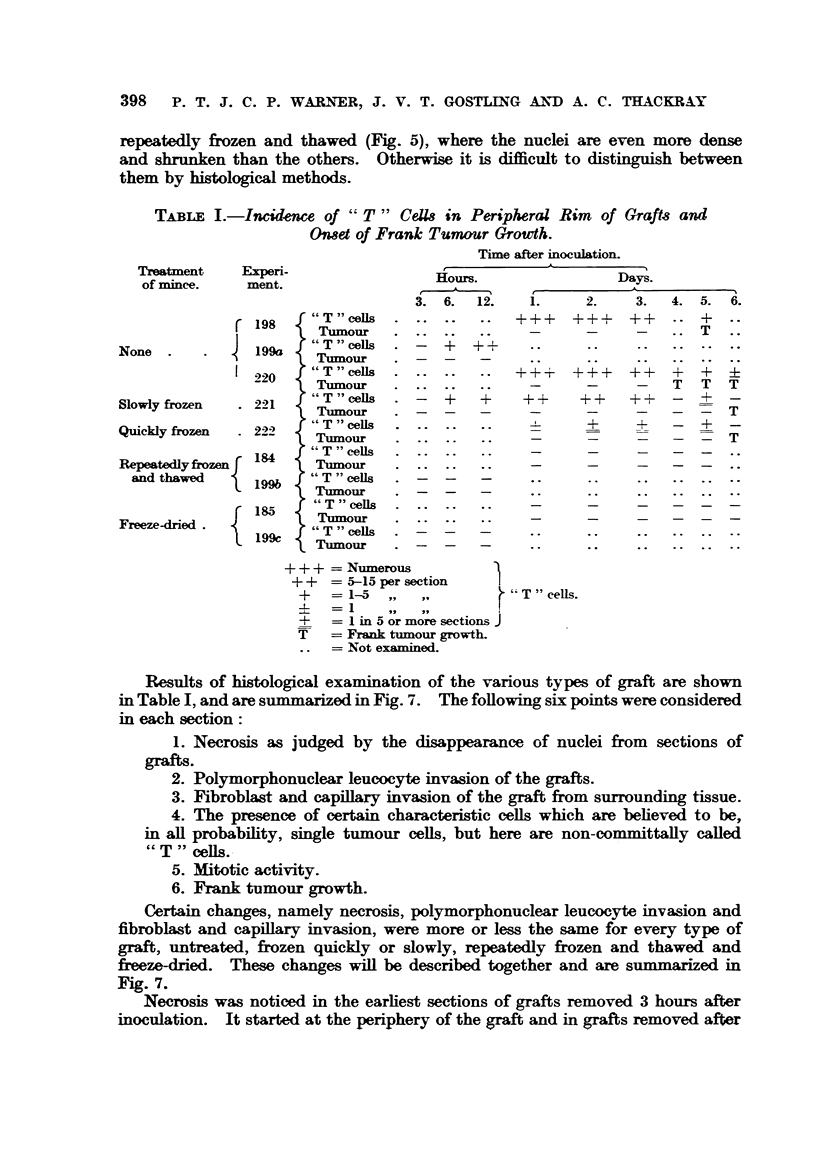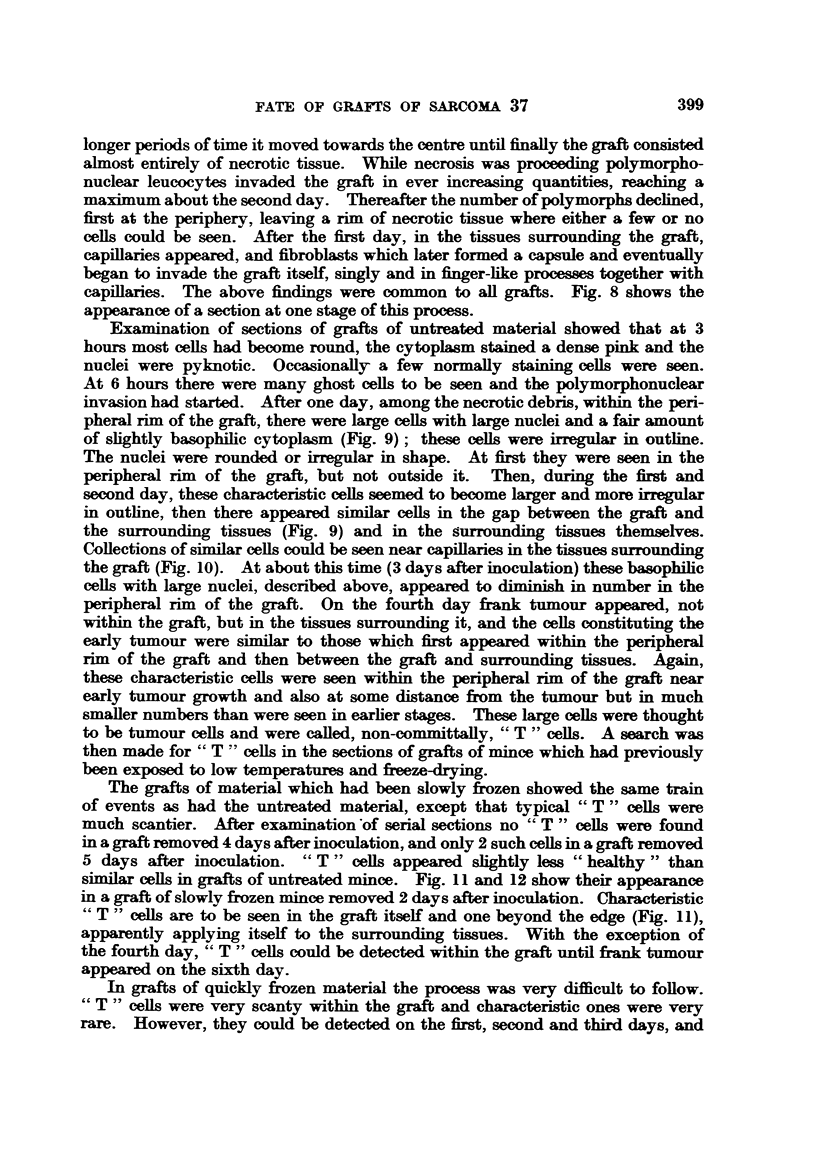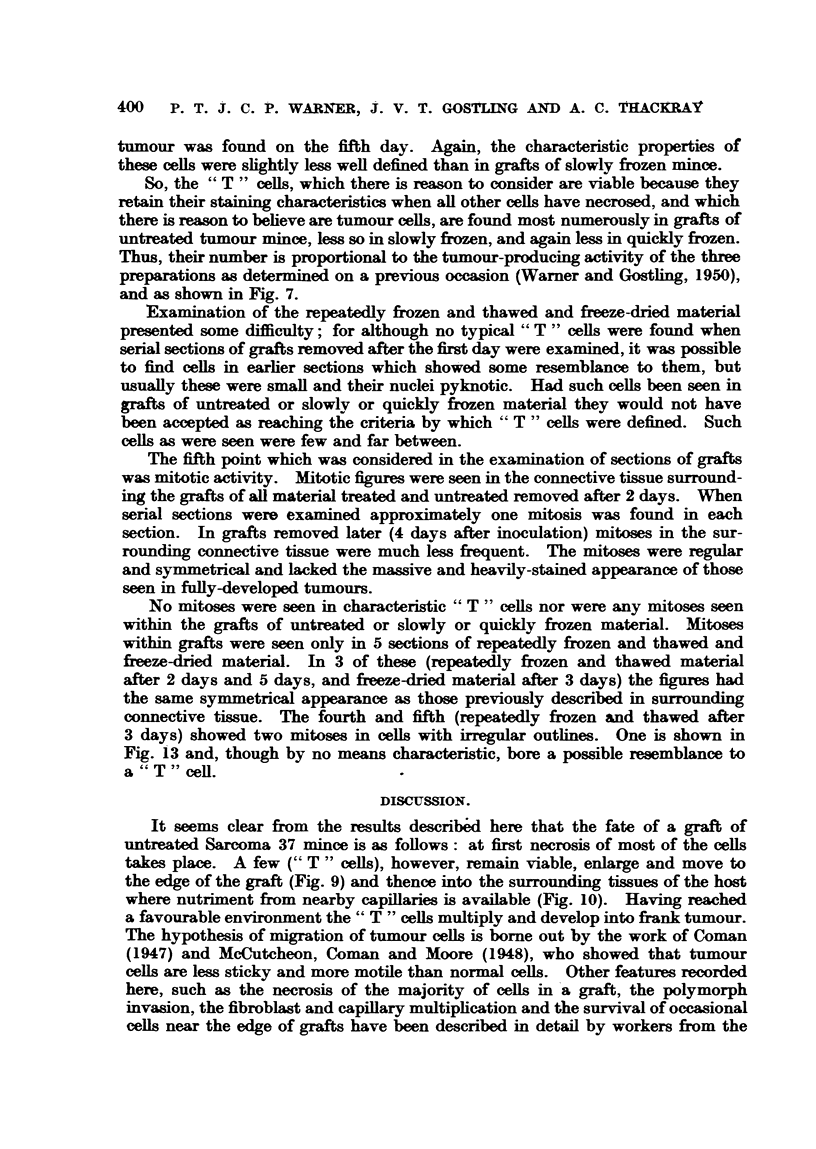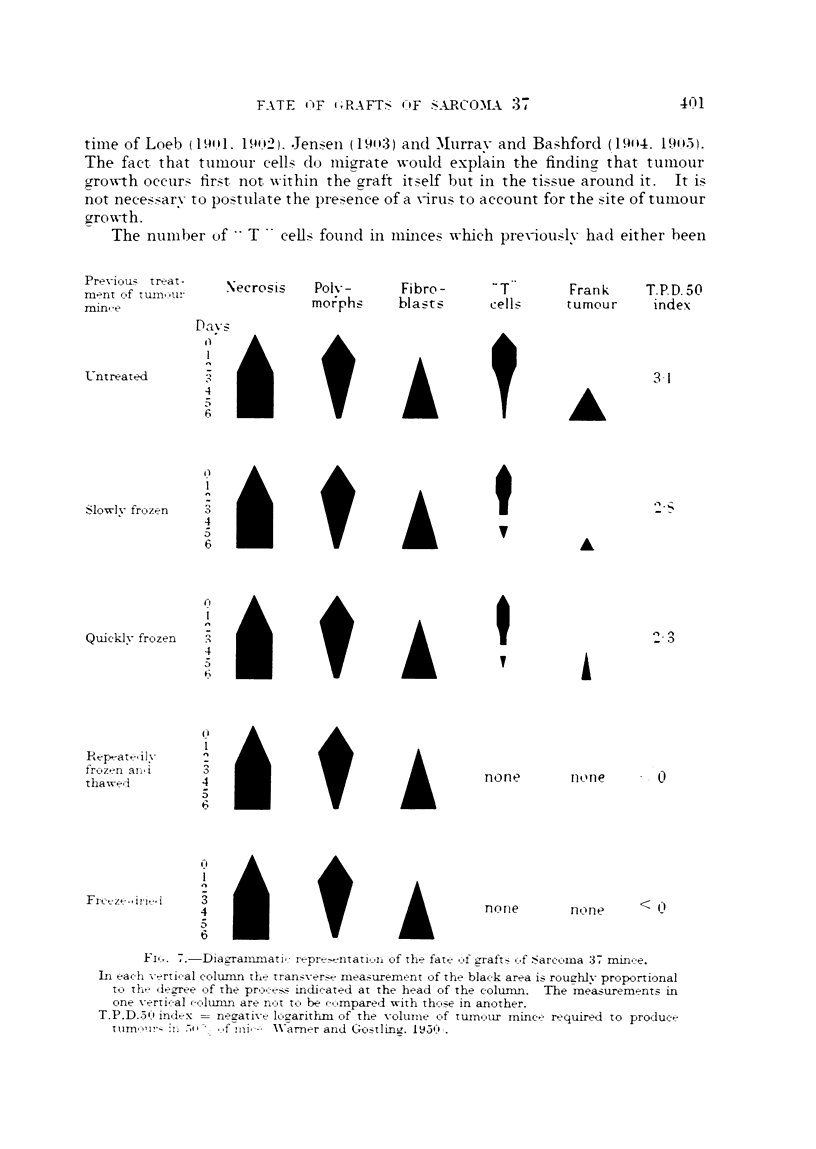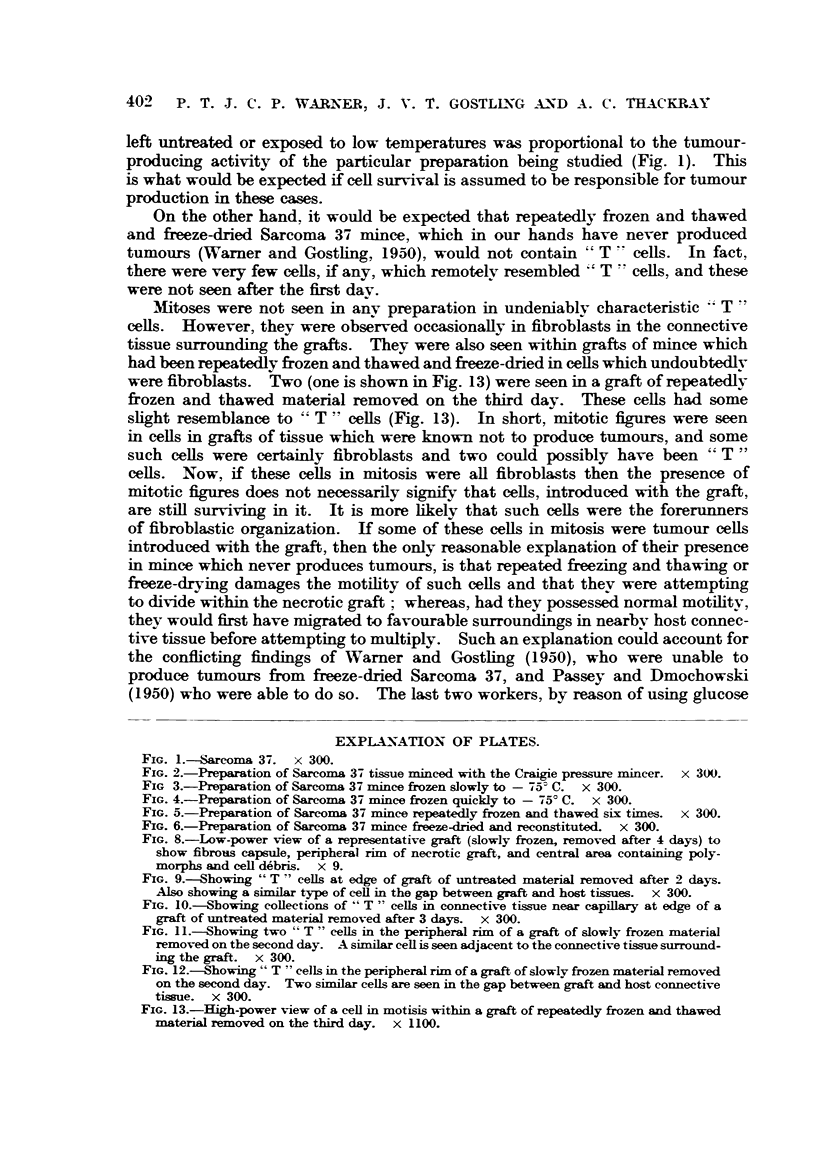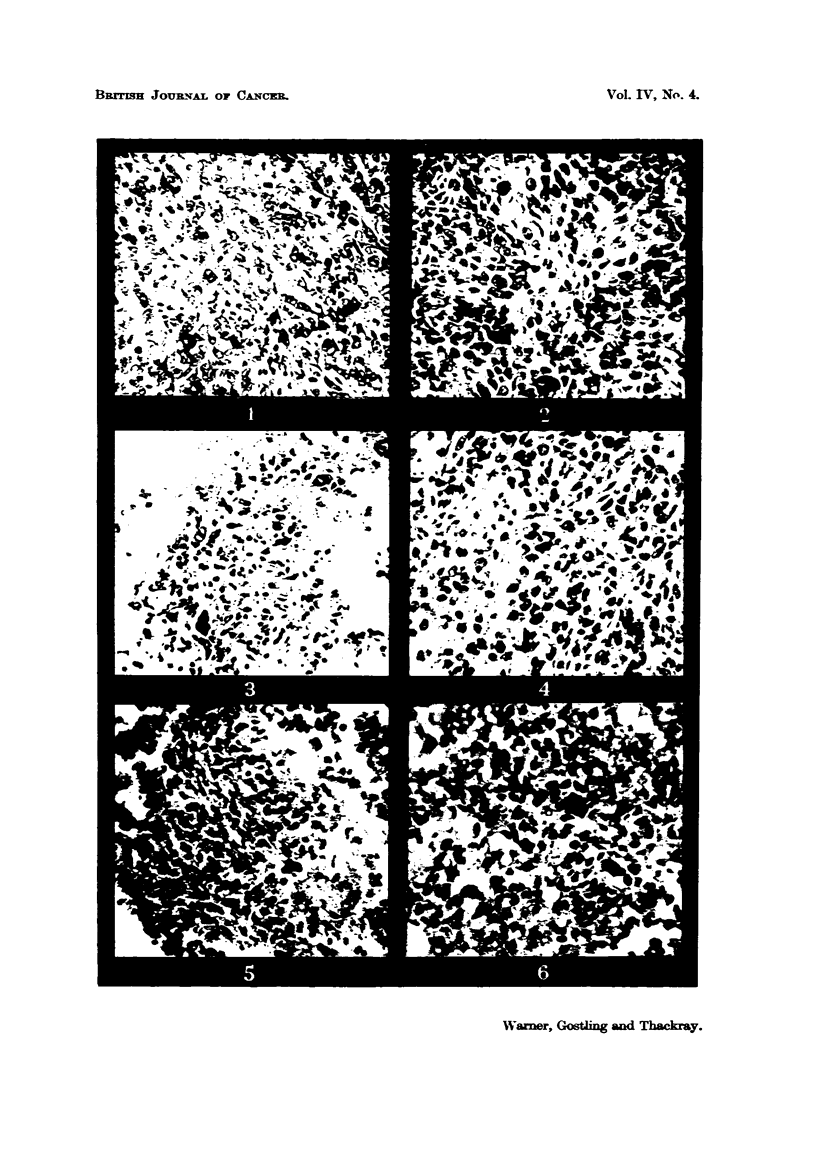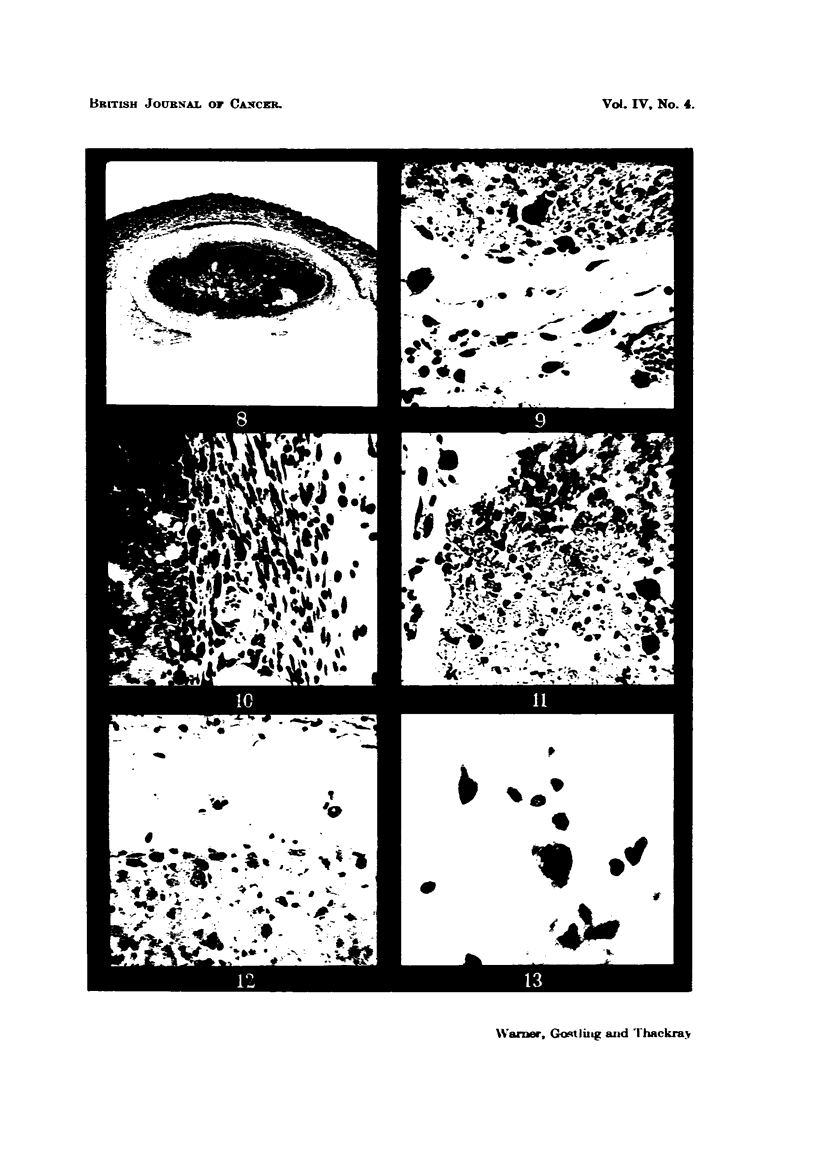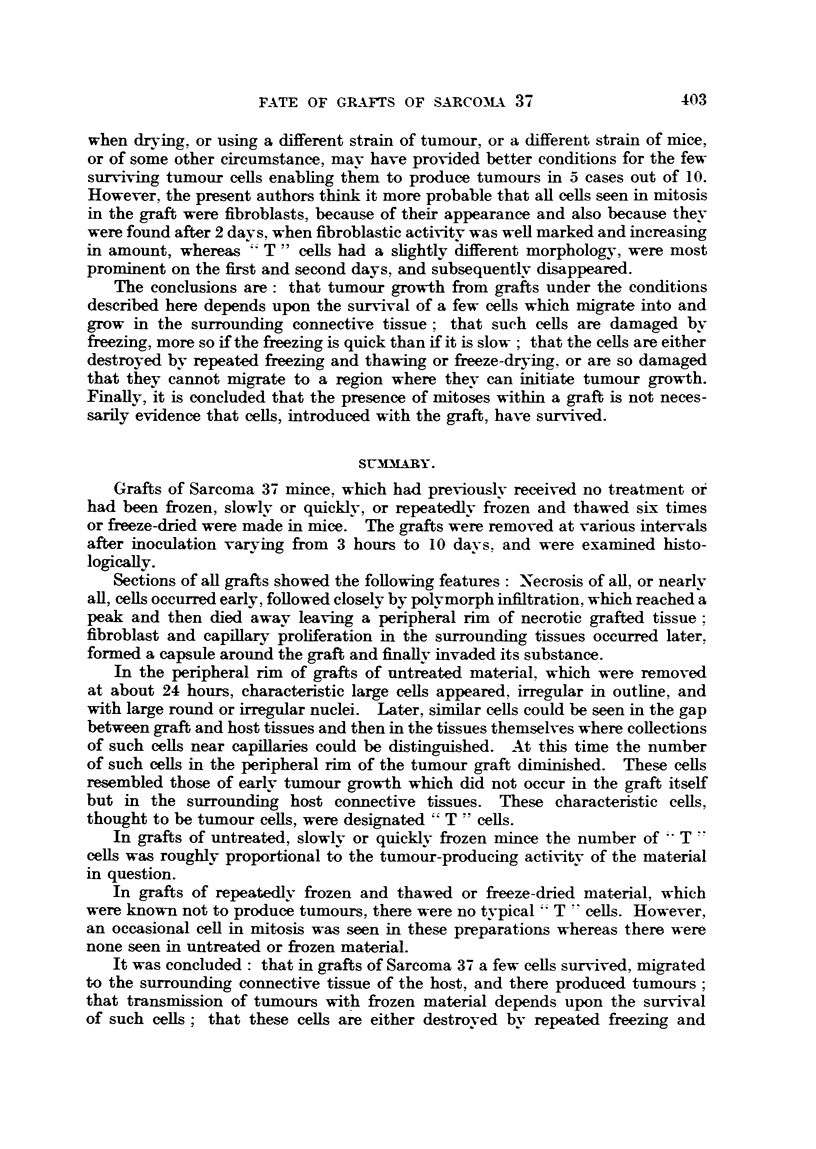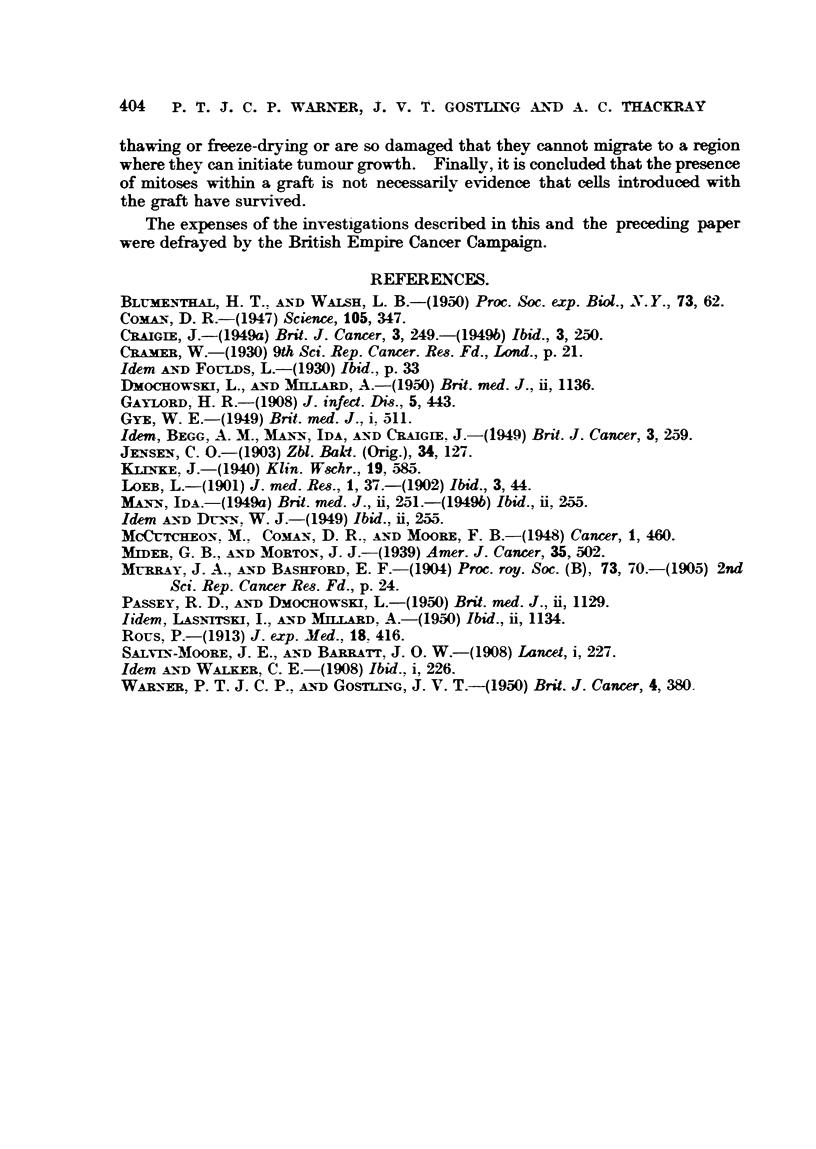# The Fate of Grafts of Sarcoma 37 Mince after Exposure to Low Temperature and Freeze-drying

**DOI:** 10.1038/bjc.1950.38

**Published:** 1950-12

**Authors:** P. T. J. C. P. Warner, J. V. T. Gostling, A. C. Thackray

## Abstract

**Images:**


### THE FATE OF GRAFTS OF SARCOMA 37 MINCE AFTER EXPOSURE TO LOW TEMPERATURE AND FREEZE-DRYING.

P. T. J. C. P. WARNER, J. V. T. GOSTLING AND A. C. THACKRAY.

*From the Bland-Sutton Institute of Pathology, Middlesex Hospital, London, W.1.*

Received for publication December 8, 1951.

NUMEROUS workers have subjected normal and neoplastic tissues to low temperatures and some to drying. Many of the reports of these workers have been referred to in a previous paper (Warner and Gostling, 1950).

Some workers have assumed or suggested that freezing and freeze-drying processes killed the cells that they were studying (Salvin-Moore and Walker, 1908; Salvin-Moore and Barratt, 1908; Gaylord, 1908; Rous, 1913; Cramer, 1930; Cramer and Foulds, 1930; Gye, 1949; Gye, Begg, Mann and Craigie, 1949; Man, 1949*a*, 1949*b*; Mann and Dunn, 1949). However, most of these workers made no attempt to determine directly whether the actual cells that they exposed to low temperatures and freeze-drying survived or not.

On the other hand, Mider and Morton (1939), using rat skin, Blumenthal and Walsh (1950), using guinea-pig thyroid, and Dmochowski and Millard (1950), using C3H and C48 mouse sarcomata, subjected tissue to low temperatures or freeze-drying and studied their behaviour, by histological methods, when grafted into animals. These workers found evidence of growth or survival of cells and mitotic activity in the grafted tissue.

Other workers have subjected tissues to low temperatures or freeze-drying and subsequently attempted to grow them in tissue culture (Cramer, 1930; Klinke, 1940; Passey, Dmochowski, Lasnitski and Millard, 1950). The results showed survival of some types of tissue, neoplastic and normal, and not of others.

In tissue culture, Cramer (1930) obtained no growth of Sarcoma 37 which previously had been repeatedly frozen and thawed, whereas grafts of the same tissue grew *in vivo*. Passey and Dmochowski (1950) dried a suspension, in glucose solution, of minced Sarcoma 37, from the frozen state. More glucose solution was added to the dried material and it was then inoculated into mice. Five out of the ten mice thus inoculated developed tumours.

A previous paper (Warner and Gostling, 1950) described experiments in which minced Sarcoma 37 which had been frozen, quickly or slowly, to a temperature of below — 75° C. was capable of producing tumours when inoculated into mice. It was also shown, contrary to Cramer (1930) and Passey and Dmochowski (1950), that if the tissue were repeatedly frozen and thawed or dried from the frozen state, no tumours resulted.

Since some workers have assumed that freezing or freeze-drying kills tumour cells and that a virus must be implicated in the transmission of tumours by tissue treated in such a way, it was considered important to study the fate of grafts of Sarcoma 37 under similar conditions.

This paper describes the results of histological examinations, at various intervals after inoculation, of grafts of Sarcoma 37 untreated and previously treated by slow freezing, quick freezing (to below —75° C. in each case) and drying from the frozen state.

### MATERIALS AND METHODS.

The material and methods used for preparing Sarcoma 37 mince, and for freezing, repeatedly freezing and thawing and freeze-drying it have been described in detail in a previous paper (Warner and Gostling, 1950) and will be briefly recapitulated here. The sarcomata were grown in albino mice, removed from them and minced in a Craigie pressure mincer (Craigie, 1949*a*). The mince was tested for bacteriological sterility. It was then either left untreated and inoculated into mice as described below, or was subjected to slow or quick freezing or repeated freezing and thawing (six times), in each case reaching a temperature of — 75° C., or dried from the frozen state by Method II (Warner and Gostling, 1950). This method of freeze-drying is practically the same as that described by Craigie (1949*b*), except that in addition secondary drying over phosphorus pentoxide was performed. Some of each of the untreated mince, that frozen by the slow and quick methods and that frozen and thawed repeatedly, was examined histologically ; the remainder was then inoculated, in doses of 1 minim, into both flanks of each one of five or more mice. The mince that had been dried from the frozen state was reconstituted by adding a volume of distilled water equal to the original volume of tumour mince subjected to the drying process. This reconstituted material was then inoculated into the flanks of mice in doses of 1 minim, exactly as described above.

At various intervals, ranging from 3 hours to 10 days, one or some of the mice were killed. The grafts were removed, fixed in Helly's fluid, histological sections were prepared and stained with haematoxylin and eosin. In some cases serial sections were prepared from the grafts.

### RESULTS.

The results are shown partly in Table I and Fig. 1 to 6 and 8 to 13, and are summarized in Fig. 7. The bacteriological sterility tests showed that the minces used in Experiments 198, 199*a*, 199*b*, 199*c*, 220, 221 and 222 were contaminated with β-haemolytic streptococci which were encountered in previous work (Warner and Gostling, 1950). A few colonies of micrococci were grown from the minces used in Experiments 184 and 185. However, there was no histological evidence of gross bacterial inflammatory processes.

The photographs in Fig. 1 to 6 show the histological appearances of the tumour mince before treatment (Fig. 2) and after slow freezing (Fig. 3), quick freezing (Fig. 4), repeatedly (× 6) freezing and thawing (Fig. 5) and after freeze-drying and reconstitution by the addition of distilled water (Fig. 6). It will be seen that the appearances of the nuclei of the minces treated by any of the methods described here differ from those of the original tumour. The nuclei are more densely staining and slightly shrunken. The differences between the minces are small (Fig. 2 to 6), except perhaps in the case of that which had been repeatedly frozen and thawed (Fig. 5), where the nuclei are even more dense and shrunken than the others. Otherwise it is difficult to distinguish between them by histological methods.

TABLE I.—*Incidence of " T " Cells in Peripheral Rim of Grafts and Onset of Frank Tumour Growth.*

| Treatment of mince. | Experiment. | | Time after inoculation. Hours. 3. | 6. | 12. | Days. 1. | 2. | 3. | 4. | 5. | 6. |
|---|---|---|---|---|---|---|---|---|---|---|---|
| None | 198 | " T " cells | .. | .. | .. | +++ | +++ | ++ | .. | + | .. |
| | | Tumour | .. | .. | .. | — | — | — | .. | T | .. |
| | 199a | " T " cells | — | + | ++ | .. | .. | .. | .. | .. | .. |
| | | Tumour | — | — | — | .. | .. | .. | .. | .. | .. |
| | 220 | " T " cells | .. | .. | .. | +++ | +++ | ++ | + | + | $\underline{\pm}$ |
| | | Tumour | .. | .. | .. | — | — | — | T | T | T |
| Slowly frozen | 221 | " T " cells | — | + | + | ++ | ++ | ++ | — | $\underline{\pm}$ | — |
| | | Tumour | — | — | — | — | — | — | — | — | T |
| Quickly frozen | 222 | " T " cells | .. | .. | .. | ± | $\underline{\pm}$ | $\underline{\pm}$ | — | $\underline{\pm}$ | — |
| | | Tumour | .. | .. | .. | — | — | — | — | — | T |
| Repeatedly frozen and thawed | 184 | " T " cells | .. | .. | .. | — | — | — | — | — | .. |
| | | Tumour | .. | .. | .. | — | — | — | — | — | .. |
| | 199b | " T " cells | — | — | — | .. | .. | .. | .. | .. | .. |
| | | Tumour | — | — | — | .. | .. | .. | .. | .. | .. |
| Freeze-dried | 185 | " T " cells | .. | .. | .. | — | — | — | — | — | — |
| | | Tumour | .. | .. | .. | — | — | — | — | — | — |
| | 199c | " T " cells | — | — | — | .. | .. | .. | .. | .. | .. |
| | | Tumour | — | — | — | .. | .. | .. | .. | .. | .. |

+++ = Numerous

++ = 5–15 per section

+ = 1–5 „ „

± = 1 „ „

$\underline{\pm}$ = 1 in 5 or more sections } " T " cells.

T = Frank tumour growth.

.. = Not examined.

Results of histological examination of the various types of graft are shown in Table I, and are summarized in Fig. 7. The following six points were considered in each section :

1. Necrosis as judged by the disappearance of nuclei from sections of grafts.
2. Polymorphonuclear leucocyte invasion of the grafts.
3. Fibroblast and capillary invasion of the graft from surrounding tissue.
4. The presence of certain characteristic cells which are believed to be, in all probability, single tumour cells, but here are non-committally called " T " cells.
5. Mitotic activity.
6. Frank tumour growth.

Certain changes, namely necrosis, polymorphonuclear leucocyte invasion and fibroblast and capillary invasion, were more or less the same for every type of graft, untreated, frozen quickly or slowly, repeatedly frozen and thawed and freeze-dried. These changes will be described together and are summarized in Fig. 7.

Necrosis was noticed in the earliest sections of grafts removed 3 hours after inoculation. It started at the periphery of the graft and in grafts removed after longer periods of time it moved towards the centre until finally the graft consisted almost entirely of necrotic tissue. While necrosis was proceeding polymorphonuclear leucocytes invaded the graft in ever increasing quantities, reaching a maximum about the second day. Thereafter the number of polymorphs declined, first at the periphery, leaving a rim of necrotic tissue where either a few or no cells could be seen. After the first day, in the tissues surrounding the graft, capillaries appeared, and fibroblasts which later formed a capsule and eventually began to invade the graft itself, singly and in finger-like processes together with capillaries. The above findings were common to all grafts. Fig. 8 shows the appearance of a section at one stage of this process.

Examination of sections of grafts of untreated material showed that at 3 hours most cells had become round, the cytoplasm stained a dense pink and the nuclei were pyknotic. Occasionally a few normally staining cells were seen. At 6 hours there were many ghost cells to be seen and the polymorphonuclear invasion had started. After one day, among the necrotic debris, within the peripheral rim of the graft, there were large cells with large nuclei and a fair amount of slightly basophilic cytoplasm (Fig. 9); these cells were irregular in outline. The nuclei were rounded or irregular in shape. At first they were seen in the peripheral rim of the graft, but not outside it. Then, during the first and second day, these characteristic cells seemed to become larger and more irregular in outline, then there appeared similar cells in the gap between the graft and the surrounding tissues (Fig. 9) and in the surrounding tissues themselves. Collections of similar cells could be seen near capillaries in the tissues surrounding the graft (Fig. 10). At about this time (3 days after inoculation) these basophilic cells with large nuclei, described above, appeared to diminish in number in the peripheral rim of the graft. On the fourth day frank tumour appeared, not within the graft, but in the tissues surrounding it, and the cells constituting the early tumour were similar to those which first appeared within the peripheral rim of the graft and then between the graft and surrounding tissues. Again, these characteristic cells were seen within the peripheral rim of the graft near early tumour growth and also at some distance from the tumour but in much smaller numbers than were seen in earlier stages. These large cells were thought to be tumour cells and were called, non-committally, " T " cells. A search was then made for " T " cells in the sections of grafts of mince which had previously been exposed to low temperatures and freeze-drying.

The grafts of material which had been slowly frozen showed the same train of events as had the untreated material, except that typical " T " cells were much scantier. After examination of serial sections no " T " cells were found in a graft removed 4 days after inoculation, and only 2 such cells in a graft removed 5 days after inoculation. " T " cells appeared slightly less " healthy " than similar cells in grafts of untreated mince. Fig. 11 and 12 show their appearance in a graft of slowly frozen mince removed 2 days after inoculation. Characteristic " T " cells are to be seen in the graft itself and one beyond the edge (Fig. 11), apparently applying itself to the surrounding tissues. With the exception of the fourth day, " T " cells could be detected within the graft until frank tumour appeared on the sixth day.

In grafts of quickly frozen material the process was very difficult to follow. " T " cells were very scanty within the graft and characteristic ones were very rare. However, they could be detected on the first, second and third days, and tumour was found on the fifth day. Again, the characteristic properties of these cells were slightly less well defined than in grafts of slowly frozen mince.

So, the " T " cells, which there is reason to consider are viable because they retain their staining characteristics when all other cells have necrosed, and which there is reason to believe are tumour cells, are found most numerously in grafts of untreated tumour mince, less so in slowly frozen, and again less in quickly frozen. Thus, their number is proportional to the tumour-producing activity of the three preparations as determined on a previous occasion (Warner and Gostling, 1950), and as shown in Fig. 7.

Examination of the repeatedly frozen and thawed and freeze-dried material presented some difficulty; for although no typical " T " cells were found when serial sections of grafts removed after the first day were examined, it was possible to find cells in earlier sections which showed some resemblance to them, but usually these were small and their nuclei pyknotic. Had such cells been seen in grafts of untreated or slowly or quickly frozen material they would not have been accepted as reaching the criteria by which " T " cells were defined. Such cells as were seen were few and far between.

The fifth point which was considered in the examination of sections of grafts was mitotic activity. Mitotic figures were seen in the connective tissue surrounding the grafts of all material treated and untreated removed after 2 days. When serial sections were examined approximately one mitosis was found in each section. In grafts removed later (4 days after inoculation) mitoses in the surrounding connective tissue were much less frequent. The mitoses were regular and symmetrical and lacked the massive and heavily-stained appearance of those seen in fully-developed tumours.

No mitoses were seen in characteristic " T " cells nor were any mitoses seen within the grafts of untreated or slowly or quickly frozen material. Mitoses within grafts were seen only in 5 sections of repeatedly frozen and thawed and freeze-dried material. In 3 of these (repeatedly frozen and thawed material after 2 days and 5 days, and freeze-dried material after 3 days) the figures had the same symmetrical appearance as those previously described in surrounding connective tissue. The fourth and fifth (repeatedly frozen and thawed after 3 days) showed two mitoses in cells with irregular outlines. One is shown in Fig. 13 and, though by no means characteristic, bore a possible resemblance to a " T " cell.

### DISCUSSION.

It seems clear from the results described here that the fate of a graft of untreated Sarcoma 37 mince is as follows: at first necrosis of most of the cells takes place. A few (" T " cells), however, remain viable, enlarge and move to the edge of the graft (Fig. 9) and thence into the surrounding tissues of the host where nutriment from nearby capillaries is available (Fig. 10). Having reached a favourable environment the " T " cells multiply and develop into frank tumour. The hypothesis of migration of tumour cells is borne out by the work of Coman (1947) and McCutcheon, Coman and Moore (1948), who showed that tumour cells are less sticky and more motile than normal cells. Other features recorded here, such as the necrosis of the majority of cells in a graft, the polymorph invasion, the fibroblast and capillary multiplication and the survival of occasional cells near the edge of grafts have been described in detail by workers from the time of Loeb (1901, 1902), Jensen (1903) and Murray and Bashford (1904, 1905). The fact that tumour cells do migrate would explain the finding that tumour growth occurs first not within the graft itself but in the tissue around it. It is not necessary to postulate the presence of a virus to account for the site of tumour growth.

The number of "T" cells found in minces which previously had either been

FIG. 7.—Diagrammatic representation of the fate of grafts of Sarcoma 37 mince.

In each vertical column the transverse measurement of the black area is roughly proportional to the degree of the process indicated at the head of the column. The measurements in one vertical column are not to be compared with those in another.

T.P.D.50 index = negative logarithm of the volume of tumour mince required to produce tumours in 50% of mice (Warner and Gostling, 1950).

left untreated or exposed to low temperatures was proportional to the tumour-producing activity of the particular preparation being studied (Fig. 1). This is what would be expected if cell survival is assumed to be responsible for tumour production in these cases.

On the other hand, it would be expected that repeatedly frozen and thawed and freeze-dried Sarcoma 37 mince, which in our hands have never produced tumours (Warner and Gostling, 1950), would not contain " T " cells. In fact, there were very few cells, if any, which remotely resembled " T " cells, and these were not seen after the first day.

Mitoses were not seen in any preparation in undeniably characteristic " T " cells. However, they were observed occasionally in fibroblasts in the connective tissue surrounding the grafts. They were also seen within grafts of mince which had been repeatedly frozen and thawed and freeze-dried in cells which undoubtedly were fibroblasts. Two (one is shown in Fig. 13) were seen in a graft of repeatedly frozen and thawed material removed on the third day. These cells had some slight resemblance to " T " cells (Fig. 13). In short, mitotic figures were seen in cells in grafts of tissue which were known not to produce tumours, and some such cells were certainly fibroblasts and two could possibly have been " T " cells. Now, if these cells in mitosis were all fibroblasts then the presence of mitotic figures does not necessarily signify that cells, introduced with the graft, are still surviving in it. It is more likely that such cells were the forerunners of fibroblastic organization. If some of these cells in mitosis were tumour cells introduced with the graft, then the only reasonable explanation of their presence in mince which never produces tumours, is that repeated freezing and thawing or freeze-drying damages the motility of such cells and that they were attempting to divide within the necrotic graft ; whereas, had they possessed normal motility, they would first have migrated to favourable surroundings in nearby host connective tissue before attempting to multiply. Such an explanation could account for the conflicting findings of Warner and Gostling (1950), who were unable to produce tumours from freeze-dried Sarcoma 37, and Passey and Dmochowski (1950) who were able to do so. The last two workers, by reason of using glucose

---

EXPLANATION OF PLATES.

FIG. 1.—Sarcoma 37. × 300.

FIG. 2.—Preparation of Sarcoma 37 tissue minced with the Craigie pressure mincer. × 300.

FIG 3.—Preparation of Sarcoma 37 mince frozen slowly to − 75° C. × 300.

FIG. 4.—Preparation of Sarcoma 37 mince frozen quickly to − 75° C. × 300.

FIG. 5.—Preparation of Sarcoma 37 mince repeatedly frozen and thawed six times. × 300.

FIG. 6.—Preparation of Sarcoma 37 mince freeze-dried and reconstituted. × 300.

FIG. 8.—Low-power view of a representative graft (slowly frozen, removed after 4 days) to show fibrous capsule, peripheral rim of necrotic graft, and central area containing polymorphs and cell débris. × 9.

FIG. 9.—Showing " T " cells at edge of graft of untreated material removed after 2 days. Also showing a similar type of cell in the gap between graft and host tissues. × 300.

FIG. 10.—Showing collections of " T " cells in connective tissue near capillary at edge of a graft of untreated material removed after 3 days. × 300.

FIG. 11.—Showing two " T " cells in the peripheral rim of a graft of slowly frozen material removed on the second day. A similar cell is seen adjacent to the connective tissue surrounding the graft. × 300.

FIG. 12.—Showing " T " cells in the peripheral rim of a graft of slowly frozen material removed on the second day. Two similar cells are seen in the gap between graft and host connective tissue. × 300.

FIG. 13.—High-power view of a cell in motisis within a graft of repeatedly frozen and thawed material removed on the third day. × 1100.

Warner, Gostling and Thackray.

8

9

10

11

12

13

Warner, Gostling and Thackray when drying, or using a different strain of tumour, or a different strain of mice, or of some other circumstance, may have provided better conditions for the few surviving tumour cells enabling them to produce tumours in 5 cases out of 10. However, the present authors think it more probable that all cells seen in mitosis in the graft were fibroblasts, because of their appearance and also because they were found after 2 days, when fibroblastic activity was well marked and increasing in amount, whereas " T " cells had a slightly different morphology, were most prominent on the first and second days, and subsequently disappeared.

The conclusions are : that tumour growth from grafts under the conditions described here depends upon the survival of a few cells which migrate into and grow in the surrounding connective tissue ; that such cells are damaged by freezing, more so if the freezing is quick than if it is slow ; that the cells are either destroyed by repeated freezing and thawing or freeze-drying, or are so damaged that they cannot migrate to a region where they can initiate tumour growth. Finally, it is concluded that the presence of mitoses within a graft is not necessarily evidence that cells, introduced with the graft, have survived.

### SUMMARY.

Grafts of Sarcoma 37 mince, which had previously received no treatment or had been frozen, slowly or quickly, or repeatedly frozen and thawed six times or freeze-dried were made in mice. The grafts were removed at various intervals after inoculation varying from 3 hours to 10 days, and were examined histologically.

Sections of all grafts showed the following features : Necrosis of all, or nearly all, cells occurred early, followed closely by polymorph infiltration, which reached a peak and then died away leaving a peripheral rim of necrotic grafted tissue ; fibroblast and capillary proliferation in the surrounding tissues occurred later, formed a capsule around the graft and finally invaded its substance.

In the peripheral rim of grafts of untreated material, which were removed at about 24 hours, characteristic large cells appeared, irregular in outline, and with large round or irregular nuclei. Later, similar cells could be seen in the gap between graft and host tissues and then in the tissues themselves where collections of such cells near capillaries could be distinguished. At this time the number of such cells in the peripheral rim of the tumour graft diminished. These cells resembled those of early tumour growth which did not occur in the graft itself but in the surrounding host connective tissues. These characteristic cells, thought to be tumour cells, were designated " T " cells.

In grafts of untreated, slowly or quickly frozen mince the number of " T " cells was roughly proportional to the tumour-producing activity of the material in question.

In grafts of repeatedly frozen and thawed or freeze-dried material, which were known not to produce tumours, there were no typical " T " cells. However, an occasional cell in mitosis was seen in these preparations whereas there were none seen in untreated or frozen material.

It was concluded : that in grafts of Sarcoma 37 a few cells survived, migrated to the surrounding connective tissue of the host, and there produced tumours ; that transmission of tumours with frozen material depends upon the survival of such cells ; that these cells are either destroyed by repeated freezing and thawing or freeze-drying or are so damaged that they cannot migrate to a region where they can initiate tumour growth. Finally, it is concluded that the presence of mitoses within a graft is not necessarily evidence that cells introduced with the graft have survived.

The expenses of the investigations described in this and the preceding paper were defrayed by the British Empire Cancer Campaign.

### REFERENCES.

Blumenthal, H. T., and Walsh, L. B.—(1950) *Proc. Soc. exp. Biol., N.Y.*, **73**, 62.

Coman, D. R.—(1947) *Science*, **105**, 347.

Craigie, J.—(1949*a*) *Brit. J. Cancer*, **3**, 249.—(1949*b*) *Ibid.*, **3**, 250.

Cramer, W.—(1930) *9th Sci. Rep. Cancer. Res. Fd., Lond.*, p. 21.

*Idem* and Foulds, L.—(1930) *Ibid.*, p. 33

Dmochowski, L., and Millard, A.—(1950) *Brit. med. J.*, ii, 1136.

Gaylord, H. R.—(1908) *J. infect. Dis.*, **5**, 443.

Gye, W. E.—(1949) *Brit. med. J.*, i, 511.

*Idem*, Begg, A. M., Mann, Ida, and Craigie, J.—(1949) *Brit. J. Cancer*, **3**, 259.

Jensen, C. O.—(1903) *Zbl. Bakt.* (Orig.), **34**, 127.

Klinke, J.—(1940) *Klin. Wschr.*, **19**, 585.

Loeb, L.—(1901) *J. med. Res.*, **1**, 37.—(1902) *Ibid.*, **3**, 44.

Mann, Ida.—(1949*a*) *Brit. med. J.*, ii, 251.—(1949*b*) *Ibid.*, ii, 255.

*Idem* and Dunn, W. J.—(1949) *Ibid.*, ii, 255.

McCutcheon, M., Coman, D. R., and Moore, F. B.—(1948) *Cancer*, **1**, 460.

Mider, G. B., and Morton, J. J.—(1939) *Amer. J. Cancer*, **35**, 502.

Murray, J. A., and Bashford, E. F.—(1904) *Proc. roy. Soc.* (B), **73**, 70.—(1905) *2nd Sci. Rep. Cancer Res. Fd.*, p. 24.

Passey, R. D., and Dmochowski, L.—(1950) *Brit. med. J.*, ii, 1129.

*Iidem*, Lasnitski, I., and Millard, A.—(1950) *Ibid.*, ii, 1134.

Rous, P.—(1913) *J. exp. Med.*, **18**, 416.

Salvin-Moore, J. E., and Barratt, J. O. W.—(1908) *Lancet*, i, 227.

*Idem* and Walker, C. E.—(1908) *Ibid.*, i, 226.

Warner, P. T. J. C. P., and Gostling, J. V. T.—(1950) *Brit. J. Cancer*, **4**, 380.